# Peer review of clinical and translational research manuscripts: Perspectives from statistical collaborators

**DOI:** 10.1017/cts.2023.707

**Published:** 2024-01-04

**Authors:** Phillip J. Schulte, Judith D. Goldberg, Robert A. Oster, Walter T. Ambrosius, Lauren Balmert Bonner, Howard Cabral, Rickey E. Carter, Ye Chen, Manisha Desai, Dongmei Li, Christopher J. Lindsell, Gina-Maria Pomann, Emily Slade, Tor D. Tosteson, Fang Yu, Heidi Spratt

**Affiliations:** 1 Division of Clinical Trials and Biostatistics, Department of Quantitative Health Sciences, Mayo Clinic, Rochester, MN, USA; 2 Division of Biostatistics, Department of Population Health, New York University Grossman School of Medicine, New York, NY, USA; 3 Department of Medicine, Division of Preventive Medicine, University of Alabama at Birmingham, Birmingham, AL, USA; 4 Department of Biostatistics and Data Science, Wake Forest University School of Medicine, Winston-Salem, NC, USA; 5 Department of Preventive Medicine, Northwestern University Feinberg School of Medicine, Chicago, IL, USA; 6 Department of Biostatistics, Boston University School of Public Health, Boston, MA, USA; 7 Department of Quantitative Health Sciences, Mayo Clinic, Jacksonville, FL, USA; 8 Biostatistics, Epidemiology and Research Design (BERD), Tufts Clinical and Translational Science Institute (CTSI), Boston, MA, USA; 9 Quantitative Sciences Unit, Departments of Medicine, Biomedical Data Science, and Epidemiology and Population Health, Stanford University, Stanford, CA, USA; 10 Department of Clinical and Translational Research, Obstetrics and Gynecology and Public Health Sciences, University of Rochester Medical Center, Rochester, NY, USA; 11 Department of Biostatistics and Bioinformatics, Duke University, Durham, NC, USA; 12 Department of Biostatistics, University of Kentucky, Lexington, KY, USA; 13 Department of Biomedical Data Science, Geisel School of Medicine, Dartmouth College, Hanover, NH, USA; 14 Department of Biostatistics, University of Nebraska Medical Center, Omaha, NE, USA; 15 Department of Biostatistics and Data Science, School of Public and Population Health, University of Texas Medical Branch, Galveston, TX, USA

**Keywords:** Biostatistics, peer review, reviewer guidance, clinical and translational science, study design

## Abstract

Research articles in the clinical and translational science literature commonly use quantitative data to inform evaluation of interventions, learn about the etiology of disease, or develop methods for diagnostic testing or risk prediction of future events. The peer review process must evaluate the methodology used therein, including use of quantitative statistical methods. In this manuscript, we provide guidance for peer reviewers tasked with assessing quantitative methodology, intended to complement guidelines and recommendations that exist for manuscript authors. We describe components of clinical and translational science research manuscripts that require assessment including study design and hypothesis evaluation, sampling and data acquisition, interventions (for studies that include an intervention), measurement of data, statistical analysis methods, presentation of the study results, and interpretation of the study results. For each component, we describe what reviewers should look for and assess; how reviewers should provide helpful comments for fixable errors or omissions; and how reviewers should communicate uncorrectable and irreparable errors. We then discuss the critical concepts of transparency and acceptance/revision guidelines when communicating with responsible journal editors.

## Introduction

The types of articles and clinical research studies evaluated and considered for publication by medical journals are broad and diverse, as are the associated statistical methods appropriate for analysis of those studies. Review of statistical methodology is a core component of the peer review process, but the statistical review process for submissions varies greatly among journals, as does the quality and depth of statistical review among individual reviewers, even within the same journal. Some journals retain biostatistics experts for peer review, although this practice is not widespread. Identification of experts in quantitative methodology and experience or expertise in the clinical or translational research domain with interest and availability to serve as a reviewer or editorial board member is a common challenge for editors [1–4]. Even when appropriate reviewers are identified, there is often little guidance provided to help the reviewer and to enable appropriate, equitable review practices. Given the diversity of reviewers and practices, the scientific community would benefit from guidance that could streamline the review process and provide a unified, structured, and systematic approach. Such guidance could provide more transparent, constructive, and consistent reviews to authors and promote robust scientific practice [2].

Many publications provide statistical guidelines for authors and research collaborators, including those published in general medical journals [5,6] or as books [7,8] and those in subject-specific medical journals [9–19]. Resources targeting statistics and quantitative methodology professionals also provide relevant guidelines[20–22]. Alternatively, many clinical and translational science journals provide guidelines on websites in the instructions for authors [23–26]. While these publications provide guidance to the authors when writing a paper and performing analyses that are reported, these guidelines are generally tailored to basic or commonly used statistical methods. Moreover, ongoing developments in quantitative methods mean that best practices are ever-changing, and analysis guidance documents may struggle to stay current. Reporting guidelines were developed to focus on transparent reporting of results, but not necessarily to provide guidance for research conduct, for randomized trials [27], observational studies [28], meta-analyses and systematic reviews [29], diagnostic/prognostic prediction studies [30–32], and preclinical animal studies [33], among many[34]. Ethical guidelines also exist for peer review but without focus on the content of a conducted review [35]. Recommendations for statistical analysis and methods reporting for authors also exist [36,37]. Taken together, these publications and reports provide comprehensive recommendations for authors but lack guidance for the peer reviewer tasked with reviewing the scientific content of a submitted manuscript and the application of quantitative methodology and biostatistics in that manuscript. While prior papers have provided recommendations for the peer reviewer of submissions to medical journals, recommendations are often specific and lack generalizability to the broader medical research community or are tailored to a narrow audience of biostatisticians with formal training in statistical methods [38]. Recommendations for the peer reviewer should consider the diversity of scientific work while maintaining consistency with ongoing and future developments in the statistical sciences. These recommendations should also be accessible to a broader community of reviewers who evaluate quantitative methods in a manuscript [39]. Further, the need exists for guidance and recommendations in communication to editors and authors to articulate statistical concerns and necessary revisions clearly.

The purpose of this manuscript is to provide guidance for reviewers who are responsible for assessing the application of quantitative methodology and is intended to complement the guidelines and recommendations for authors previously described. We describe key components of clinical and translational science research manuscripts that require attention to the statistical methodology or that have considerable implications for statistical methods. These include study design and hypothesis, sampling (selection of the study subjects), interventions (for studies that include an intervention), measurement (assessment of study variables including outcome measures), statistical analysis methods, presentation of the study results, and interpretation of the study results. For each component, we describe what reviewers should look for and assess; we offer recommendations for how reviewers should provide helpful comments for fixable errors or omissions; and we describe how reviewers should communicate uncorrectable and irreparable errors. We next provide a checklist summarizing what to look for and assess when reviewing a manuscript (Table 1). We then discuss the critical concepts of transparency and acceptance/revision guidelines when communicating with responsible journal editors.

**Table 1.** Checklist guide of items to consider in biostatistical review of clinical and translational manuscripts

**Objective, hypothesis, and design**
Assess whether research aim is coherent, sensible, and statistically evaluable Evaluate whether a proposed study design supports the research objective
**Sampling**
Assess whether sampling framework is described well Evaluate whether subsequent methods align with data collection Evaluate sample size justification Evaluate documentation for appropriateness of selected sample
**Intervention**
Assess when and how the intervention was applied Assess timing of the intervention relative to outcome or endpoint assessments
**Measurement**
Assess whether adequate descriptions of measurements are provided Assess how missingness arose in the study and if analysis of missing data is appropriate Assess whether statistical analyses reflect the nature of the measurement of the variables Evaluate whether the methods sufficiently allow for assessment of measurement error
**Analysis methods**
Evaluate description of statistical methods for reproducibility Determine whether assumptions for use of specific statistical methods are described and assessed
**Presentation**
Ensure consistent and accurate presentation throughout manuscript Ensure tables and figures have descriptive title, are clear, and self-explanatory
**Interpretation**
Evaluate whether interpretation of results reflects what can be inferred from study results Assess whether limitations of the study are thoroughly noted Assess whether there is necessary context for conclusions

## Objective, hypothesis, and design

### Evaluation

The study objective or aim, and study design set the framework for a scientific manuscript. Reviewers must first assess whether the research objective or aim is coherent, sensible, and statistically evaluable; subsequently, the reviewer evaluates whether a proposed study design supports the research objective. The research objectives are defined inclusively to encompass comparisons of interventions or therapies (randomized or observational), epidemiological and etiological studies, prediction and diagnosis objectives, mechanistic studies, and more.

### Suggestions for Revision

Fixable flaws from a study design perspective generally relate to a lack of clarity in the description of the design or a poor description of the objectives. Comments to the authors’ revision may focus on requests for clarification or additional detail, or revisions to wording to clarify aims. For example, studies that specify a testable hypothesis are typically described as the statistical alternative hypothesis. That is, authors might hypothesize that a treatment causes a difference in outcome, on average, between those treated and untreated. This corresponds to the statistical alternative hypothesis whereas the null hypothesis is “no difference” between groups. On occasion, authors will incorrectly describe the hypothesis using language corresponding to the null hypothesis of the statistical methods used to analyze the data, providing a hypothesis that “groups will be similar.” This may necessitate a comment suggesting a change in analysis methods or a change in how hypotheses are described. This is because statistical tests in the frequentist framework either reject or do not reject the null hypothesis and the colloquial phrase: absence of evidence to reject the null hypothesis is not evidence of the null hypothesis.

### Irreparable Issues

A study design that is not aligned with the research objective is generally not fixable since this would require a new study to be conducted using a more appropriate design. For example, the aim of an epidemiological study might be to evaluate the association between an exposure and outcome. If the exposure is measured and ascertained after the outcome, conclusions may be muddied by reverse causation and other logical flaws. When the study design does not provide a statistically valid approach to address the objectives or aims, a recommendation of rejection can be provided to the editor with a well-reasoned description of the concern to the authors.

### Unhelpful Comments

Reviewers should be cautious in distinguishing between inappropriate study designs and designs that are adequate but perhaps not the reviewer’s preferred approach. For example, a well-reasoned, -analyzed, and -reported observational comparative effectiveness study may be a sound design advancing the science of the research objective despite not being a gold-standard randomized trial. In general, there may be multiple statistically principled study designs that can address a given research question; thus, comments to authors should not suggest a reviewer-preferred approach or recommend rejection of the manuscript if the existing approach is still valid and interpreted correctly.

## Sampling

### Evaluation

Researchers refer to the set of study subjects used in a research objective as a “sample” whereas “population” reflects a broader group to which the conclusions or results of the study are applicable. Reviewers should typically expect a description of how researchers planned to collect the sample of data, including (i) description of the population of interest, (ii) framework for acquiring a sample, and (iii) justification of sample size. The population of interest is typically described by inclusion and exclusion criteria, and the reviewer should ensure that these criteria align with the study aims. In clinical and translational science, samples are most often driven by convenience such as electronic medical records from a particular clinic, subjects enrolled and providing informed consent at a participating site or sites, mice or animals bred by a single supplier, or tissue and serum from patients seen at a hospital or clinic [40]. Stratified sampling or clustered sampling may be used in some studies. A reviewer must first assess whether this sampling framework is described well but also evaluate whether subsequent methods align with data collection and whether limitations of a sampling framework are appropriately acknowledged [41]. In observational data analyses, the framework for sampling may require a description of whether data are cross-sectional, cohort, or case-control, for example, with appropriate justification for whether that data can address the aims of the study. Missing data are not a sample characteristic but rather a Measurement concern, while complete case analysis is Analysis Methods decision or choice rather than a characteristic of the sample; critique of missing data is subsequently addressed in those sections. Finally, sample size should often be justified before data are collected (*a priori*), such as evaluating statistical power [42–45], anticipated Bayesian posterior distributions [46,47], or relevant metrics for prediction model development [48–51]. Whether a numeric calculation such as this is described or a qualitative justification is given, reviewers should assess the robustness of this justification.

After the study is conducted, the characteristics of the selected sample should be summarized to facilitate an assessment on how the sample might differ from the target population, thus allowing for an evaluation of the generalizability and transportability of the results. This evaluation may be assisted by a table showing summary statistics for subject characteristics. *Generalizability* and *transportability* reflect whether results obtained in the sample are reasonably applied to other subjects in the population [52]. Biased representation of the sample to the target population or poor generalizability – such as a convenience sample lacking racial diversity present in the population, a tertiary referral hospital with more complex cases than a typical hospital, or a preclinical study using only male mice to study a condition that affects both sexes – may unknowingly affect the interpretation of the results. The lack of generalizability or transportability may exacerbate inequalities and lead to poor medical decision-making – even harm [52,53]. Thus, the reviewer should critically evaluate whether the sample of data reasonably reflects the population in which the intervention, statistical model, or characteristic will be applied.

### Suggestions for Revision

Reviewers should first ensure that sufficient information is provided to assess potential biases. The impact of the sampling process, even with major limitations that make the sample a “convenience” sample, can be mitigated in some contexts with appropriate experimental designs or statistical analysis methods. Otherwise, reviewers should look for a thorough discussion of the implications of the sampling process. If any of the items mentioned above (target population, sample size determination, sample selection, and representativeness of the sample discussion) are missing, the reviewer can suggest the authors provide such information to ensure the study was rigorously conducted.

If a sample size justification was not prepared prior to the analysis, the reviewer should request a revised method section with detail how the existing sample size was determined and a quantitative calculation provided but clearly denoted as being done after the analysis was conducted. *Post hoc* or *observed* power is never an appropriate request from the reviewer, but the reviewer may request power to detect a “small effect” or calculate the difference between groups that the sample size provides 80% or 90% power to detect [54]. When sample size is fixed due to external constraints such as a retrospective data source or limited population such as a rare disease setting, a similar quantitative sample size justification may be provided along with the qualitative description of the constraints.

### Irreparable Issues

Reviewers should first give authors an opportunity to explain why the particular sampling approach was used and address limitations. We note, however, that studies that employ a sample with poor generalization to the population or exclude a subset of the population are not generally fixable through peer review. Such instances may include studies that were performed on a single sex, gender, race/ethnic group, or age group when the disease/virus/predicament under study is applicable to many.

### Unhelpful Comments

It is generally not helpful to suggest that the authors obtain new data under an alternative sampling approach. Unless the manuscript is describing future planned research, at the time a manuscript is under review, data have already been collected and the reviewer should respect contributions of animals or human subjects or samples to the research when possible. Instead, reviewers should require a robust discussion of limitations or future research needs.

## Intervention

### Evaluation

Not all clinical and translational research involves the study of interventions, therapies, or treatments applied to samples. Rather, some studies assess etiology of disease, ability to diagnose conditions, or predict future outcomes, for example. However, those who evaluate interventions, treatments, or therapies will be expected to explain when and how the intervention was applied, with a level of fidelity that allows readers or future researchers to replicate the results [55]. In trials that prospectively prescribe intervention (broadly encompassing single arm, crossover, cluster-randomized, parallel arm randomized, and others), this intervention should be prescribed *a priori* in the study protocol and subsequently described in the submitted manuscript. In analysis of observational studies with retrospective or prospective data collection, authors should include a comprehensive description of how the intervention is defined and data to assess the effects of the intervention were obtained.

The timing of intervention relative to collection of outcomes should reflect the potential biological mechanisms of action so that outcomes are not ascertained too early nor too late relative to the application of the intervention. In prospective studies, outcome assessments can be controlled at observation timepoints with biological relevance. In retrospective studies, the reviewer must carefully assess whether authors’ collection of these data could bias the study, such as a survivorship bias or immortal time bias [56].

### Suggestions for Revision

Even if the intervention is well-described intervention, there may be a need to recommend a robust discussion of study limitations. Additional suggestions may depend on the study design. A reviewer, for example, might suggest authors develop a Directed Acyclic Graph [57] with subject expertise to assess potential confounding in an observational comparative effectiveness study. While proper randomization and intention-to-treat principles prevent bias due to confounding, adjustment for prognostic variables in randomized interventional studies should be similarly pre-specified [58]. Crossover trials [59–61], before-after implementation studies [62,63], cluster-randomized trials, and others [64] each have implications that reviewers should consider to ensure authors have provided an adequate description of the intervention and limitations of the intervention applicable to the specified design.

### Irreparable Issues

In retrospective studies, authors may be limited by available data and granularity of that data. For example, an administrative database may document interventions applied during an intensive care unit admission without recording specific timing of the intervention. A study to evaluate outcomes also measured during the intensive care unit admission could be unable to determine whether the timing of intervention relative to outcome reflects biological plausibility. Reviewers would need to rely on subject-matter knowledge to understand whether the data can plausibly evaluate the objective or hypothesis, and whether concerns can be mitigated through identified limitations or whether the data are not adequate to evaluate for the study objective.

### Unhelpful Comments

Suggested changes regarding how the interventions were implemented are not helpful in studies that are underway or complete. Rather, the reviewer should give constructive comments or suggestions to improve the description of the intervention and suggest corresponding limitations.

## Measurement

### Evaluation

Measurement refers to both the collection or recording of data, data quality, and data fidelity. Measurement may reflect the timing of data collection such as a visit schedule in a prospective trial. This domain also includes whether data are missing, and the extent and reasons for missing data. Measurement also reflects how data are documented in source or raw data for the study, recorded for later analysis as analysis data, and whether such data are either continuous, interval, count, binary, nominal categorical, or ordinal categorical. Time-to-event data include a binary or categorical event or state status and continuous or interval time. Sufficient descriptions of measurements are necessary for the reader to evaluate data presented and allow for reproducibility in future research.

The reviewer should assess how the missingness arose in the study and whether treatment of missing data is appropriately handled and discussed in the manuscript. Longitudinal studies should address missingness along the entire measurement time course. This should be examined at both the individual variable level (how many subjects are missing the variable information) as well as the overall study level (how many key variables have missing data). When missing data are present, statistical analysis methods should be described and limitations and assumptions of that method should be defined. Notably, missing data does not appear under Sampling. While complete case analyses are common in clinical and translational research, this is a measurement and analysis choice by the analyst rather than a sample or population characteristic; therefore, missing data should generally not be an inclusion or exclusion criteria.

Statistical analyses should reflect the nature of the measurement of the variables; for example, continuous variables should be analyzed with methods appropriate for continuous data. Continuous data should not be discretized or dichotomized unless there is a clear mechanistically based rationale, or the categories are well accepted and validated externally. When considering statistical inference, in general, discretization of continuous data does not represent a biologically plausible relationship. On the other hand, ordinal categorical data (examples include tumor stage, Likert scale survey response) should generally not be analyzed as continuous data. An additional consideration includes assessment of measurement error or misclassification error. The reviewer should evaluate whether the methods sufficiently allow for assessment of measurement error and, if applicable, whether statistical analyses are appropriate and whether limitations are discussed.

### Suggestions for Revision

A thorough description of missing data and measurement is often lacking in research manuscripts. Reviewers should request justifications for decisions based on measurement of the data. Two common examples include use of complete case analysis without consideration of missing data mechanisms and arbitrary categorization of continuous data. Both may lead to biased interpretation or loss of precision [65–70] and, if not fixable through revision, authors should be expected to provide discussion of the limitations applicable to these approaches.

### Irreparable Issues

Incorrect assessments or measurements of the source data are rarely uncorrectable. Indeed, many statistical methods have been developed to explain signals in the presence of error and uncertainty in measurement.

### Unhelpful Comments

When data are collected poorly or poorly measured at the source data, for example, a researcher using categories of age in decades (40-49, 50-59, etc.) rather than integer or more specific continuous age, it may not be feasible for the researcher to revisit data collection. As noted in *Suggestions for Revision*, the reviewer may request revision if applicable to the available source data. Beyond that, measurement issues might be addressed through statistical analysis and also with robust discussion of limitations and future research needs.

## Analysis Methods

### Evaluation

Statistical analysis methods should be described in detail so that another researcher with adequate training could replicate the analysis and obtain the same results if given the same dataset [36,71]. The analysis methods description should tie each aim or hypothesis described in the introduction to specific analysis methods. When applicable, reviewers need to consider implications of multiple comparisons corresponding to more than one hypothesis [72]. Reviewers should expect analysis methods in the main manuscript methods section, but in those circumstances when the complexity of analysis is far greater than the typical study, a thorough and detailed methods section may be included as supplementary material if allowed by the journal.

### Suggestions for Revision

When analysis methods are vague, reviewers should request specification and detail in a revised manuscript that allows for replication of the analysis. The reviewer should ensure that analysis methods not commonly used in the subject-matter discipline are described and cited.

Analysis methods should describe statistically valid and appropriate methods based on the Sampling, Intervention (if applicable), and Measurement of data to address study Objectives. If there are discrepancies, the reviewer can suggest that certain outcomes or objectives might be better analyzed using alternative approaches. Multiple statistical methods may often be valid, but there are potential tradeoffs that the researchers must consider; for example, some methods may carry fewer assumptions about the data and analysis model, some may be more statistically powerful in limited scenarios, or there may be nuanced differences in interpretation while still aligned with the overarching objective. The reviewer should ensure assumptions of statistical methods are described and assessed where appropriate and that the description of the methods used appropriately considers tradeoffs.

### Irreparable Issues

Errors in the statistical analysis methods are rarely unfixable; however, analysis flaws can uncover major concerns with Sampling or Measurement of data.

### Unhelpful Comments

The reviewer should be open to alternative analytic approaches. Consider, for example, that a binary outcome can be regressed on covariates using logistic regression, log-binomial or Poisson regression, linear probability models, or other approaches – estimating an odds ratio, risk ratio, or risk difference, respectively. While each method requires that different assumptions are satisfied, there are many situations where more than one approach is valid and others in which one or more approaches may not be valid. The reviewer should evaluate the appropriateness of the analysis method for the study aim, keeping in mind that there may be more than one effective approach. Reviewers can ask authors to provide clear justification, including an evaluation of whether the assumptions required for the proposed methods are well-described before concluding that the methods used are unacceptable.

Pre-specified analysis plans are commonplace in prospective interventional trials and are also strongly advised for observational research [73]. When possible, reviewers should respect the plan and avoid potential biases in selecting methods based on observed data. If pre-specified plans are inadequate, reviewers may suggest post hoc secondary analyses to supplement pre-specified methods. In rare instances, pre-specified plans may represent invalid approaches and reviewers may need to suggest only the post hoc analysis be reported with a clear rationale for readers.

## Presentation

### Evaluation

Presentation includes summaries of the sample and analysis results in the text, tables, and figures. The presentation of results should follow directly from the Analysis Methods. For each analysis method described, there should be a summary of the corresponding result. The reviewer should ensure consistent and accurate presentation in the abstract, body of the manuscript, and in tables and figures. Variables, descriptions, and numeric results should be consistent throughout the manuscript. Tables and figures should have a descriptive title and, with accompanying footnotes and captions, should be clear and self-explanatory.

### Suggestions for Revision

A good table or figure can convey a large amount of high-quality data whereas a poor figure can send mixed messages. The reviewer can make specific suggestions or point toward existing resources providing robust guidance for data presentation for authors [16,74]. The reviewer should assess whether tables and figures are effectively formatted to interpret data for objectives and hypotheses and that presentation of results retains focus on the objectives. As an example, suppose a study evaluates an effect of randomized treatment vs placebo on an outcome with assessment of the outcome variable before treatment/placebo and after. A poor presentation would draw the reader’s attention to pre-post differences within each group which may be similar in both treatment and placebo study arms. Instead, the reviewer should request organization of results focusing on how treatment (vs placebo) affects the post-treatment/placebo outcome using an appropriate design and statistical method.

A focused presentation of results also suggests the reviewer should recommend removing ancillary data that are not directly related to the objectives or hypotheses. A study that develops a multivariable prediction model following applicable reporting guidelines [30] could provide a table of coefficients that can be used to output prediction, but may not need to include relative effect measures such as odds ratios with confidence intervals that are not relevant to the objective of obtaining a prediction. Similarly, if the objective is to estimate the association between an exposure and outcome adjusted for potential confounders in an observational study [57], association estimates for each confounding variable do not need to be reported. Rather, retaining focus on the exposure of interest only will help readers avoid improper inference such as the T*able 2 fallacy* [75]. Reviewers should be skeptical of extraneous statistical tests and results unrelated to study hypotheses. For example, a table of baseline characteristics in a randomized trial should not include hypothesis testing comparing pre-randomization variables by randomized arm [58], whereas in observational research such comparisons are valid but often unnecessary and irrelevant to the study objectives.

### Irreparable Issues

Presentation errors are rarely unfixable with a revision. However, issues identified when reviewing results of a manuscript may uncover additional concerns with Sampling, Measurement, or Analysis Methods that necessitate further suggestions.

### Unhelpful Comments

Problematic tables, figures, and results text require clear and constructive comments. Vague comments that critique the presentations without suggestions about how this could be improved are insufficient.

## Interpretation

### Evaluation

In the review of the interpretations of statistical analyses, there are three primary features that a reviewer should assess in a manuscript. The first is that interpretations of results should appropriately and precisely reflect what can be inferred directly from study results. For example, generalizations should only be made to the population that is reflected by the sample. The second fundamental feature of interpretation is that limitations of the study should be thoroughly noted. Reviewers should evaluate whether assumptions or incomplete knowledge that impact the data or conclusions are disclosed, including potential impacts of missing data or confounding. The limitations inherent in the data (*e.g*., missingness, surrogate variables, potential biases, etc.) should be clearly described. Reviewers should expect limitations in the data and analyses to be related with implications for interpretation of the results, rather than simply a list of limitations. When the likely impact is differential, the direction should be noted. Finally, assessing interpretation in the manuscript evaluates whether there is necessary context for conclusions. For example, the difference between statistically significant and clinically/practically significant results or the difference between confirmatory and exploratory results may need to be addressed.

### Suggestions for Revision

Since interpretation is particularly fixable at the review stage, reviewers have an opportunity to provide meaningful feedback to improve a manuscript. Critiques of interpretation are most helpful when they are specific rather than general. For example, suppose that authors interpret a null finding from a traditional hypothesis test of superiority as indicating that there is evidence of no association or difference. It would not be helpful for a reviewer to vaguely state “Interpretation of hypothesis tests are not correct.” Instead, the following critique would be more helpful: “For hypothesis tests, statistically nonsignificant results should be interpreted as providing insufficient evidence of an association, rather than as providing evidence of no association.”

### Irreparable Issues

Rarely will errors in interpretation be unfixable for a manuscript under review. It is possible for errors in interpretation to be inaccurate (rather than accurate but inappropriate), but these errors can usually be fixed through revision.

### Unhelpful Comments

Reviewers should recognize that communication is highly individual; just because authors interpret results differently than the reviewer does not necessarily make the authors’ interpretation incorrect or inappropriate. Reviewers may benefit from assessing their own critiques of interpretation by asking themselves whether the issue they are commenting on is truly incorrect or inappropriate, or whether it is just a difference in style.

## Discussion

The inclusion of statistical reviewers has been shown to improve quality of published manuscripts [76–79], but adoption of specialized review by individuals with expertise and training in statistics and quantitative methodology in medical research journals is lacking [80–82]. Review of quantitative methodology often falls to subject-matter experts with varied competency in statistical methods, sometimes with expertise in only a narrow range of statistical methods specific to their research program [39]. Recommendations for statistical review herein can be applied by expert biostatisticians and epidemiologists, as well as subject-matter practitioners with additional statistical training or competencies in quantitative methodologies.

### Recommendations and Communication

Reviewers must score and communicate recommendations to the responsible editor. These recommendations generally include (i) comments and suggestions for the authors, (ii) private comments to the editors, and (iii) a recommendation to the editors. Ultimately, the responsible editor will collect comments from multiple reviewers, consider private comments and recommendations, and make a decision on the manuscript to either accept the paper, request a revision of the paper to address reviewer concerns, or reject the paper. Prior sections have focused on how reviewers can assess core components of a clinical or translational research manuscript and provide comments to authors guiding revisions. In private comments to the editors, the reviewer should articulate any potential conflicts of interest as well as any limitations to their review. Statisticians and methodologists are rarely expert on all statistical methods and may be asked to review manuscripts that use unfamiliar methods. Similarly, subject-matter experts with limited statistical training may often review and provide recommendations related to statistical methods. In private comments to the editor, reviewers should acknowledge their own limitations. Invitations to review do not always include sufficient detail to make a determination about methodological expertise up front, so providing these comments to the responsible editor can allow them to seek additional expert opinions as needed.

Editors also request, in addition to written comments, that reviewers make a recommendation for the manuscript: accept, revise and resubmit, or reject (or variations of these). The statistical reviewer should consider recommending acceptance when there are no concerns about the study conduct or conclusions – either from the statistical review as described previously or with respect to subject-matter concerns. A recommendation to the editor to reject a manuscript must include significant justification that there are major errors in the paper and those errors are unlikely to be fixed through revision (*irreparable issues*). Finally, manuscripts with flaws identified, but not irreparable, should be recommended for revision. Some journals subclassify revisions as minor or major based on how substantial revisions will need to be and how those revisions are likely to impact the overall messaging of the manuscript. Minor revisions are unlikely to require changes to the underlying data or analysis and comments to the authors may request changes that are editorial in nature. Major revisions will include comments to the authors that request significant changes to statistical methods and re-analysis of the data.

### Responsibilities

Statistical reviewers should make recommendations based on best practices in the field of collaborative biostatistics. Reviewers often read manuscripts that use incorrect or inappropriate methods, or poor study descriptions on the basis that prior papers have done so [71]. Some concerns may have little impact on conclusions, but the reviewer has responsibility to reduce propagation of incorrect methods. Inappropriate methodology with minor impacts on overall study conclusions can be addressed via comments and requests for minor revisions.

At the same time, the statistical reviewer must be flexible and adaptive to novel approaches or changes in best practices as methodologies evolve. It is critical for the reviewer to avoid dogmatic comments, especially those that suggest specific approaches or methodologies on the basis that prior papers have used them. When reviewers are not confident that suggestions reflect best practices, they may refrain from commenting on authors, make comments expressing uncertainty to the editor, or provide comments to the author that acknowledge their own limitations and uncertainty. Finally, reviewers should be open to rebuttals that do not implement those suggestions in a revised manuscript if authors can defend that alternative approaches represent valid and correct statistical approaches.

When possible, reviewers should consider implications of pre-specified study design and statistical analysis. Pre-specification avoids biases that may arise when making design and analysis choices based on observation of intermediate results. Reviewers should consider review of those pre-specified plans and whether authors adequately adhered to those plans. This includes review of clinicaltrials.gov registration for clinical trials.

### Limitations of Statistical Review

Manuscript reviewers are tasked with evaluating whether the described methods are appropriate, and whether results are presented and interpreted correctly, but journals rarely require submission of data and programing code. As such, statistical reviewers cannot be accountable for evaluating whether implementation such as programing or software implementation was correct. In contrast with grant review or study section review [83–86], manuscript reviewers are not positioned to critique the credentials or training of the manuscript authors. The reviewer should not suggest authors “consult with a statistician” or make comments based on degree or position/appointment of authors. Rather, it is incumbent upon the authors to revise according to the objective and best practices-based comments made by the reviewer. This does not reflect a “free pass” or reduced criteria applied to those manuscripts without a biostatistician or methodologist among coauthors, but instead reflects varied approaches available for authors to implement revisions leading to pscientifically valid approach and conclusions.

### Future Directions

Guidance provided in this paper represents an opportunity for training new clinical and translational science researchers, including biostatisticians and non-biostatisticians alike. Service and national recognition achieved through exemplary peer review and editorial board participation are favorable for consideration of faculty promotion and tenure [87,88]. Thus, education on the process of statistical review may improve not only the submitted clinical and translational research papers, but benefits may extend to readers and trainees learning to improve their review skills.

## Conclusions

Reviewers rely on adequate descriptions of the analysis to provide a critique and as such, published poor-quality research is the fault of authors rather than reviewers and editors. Nonetheless, reviewers are asked to review study design, statistical methods, and presentation and interpretation of results, and can provide meaningful critiques that can improve statistical reporting and analysis in medical research. Recommendations provided in this paper are accessible to a wide audience of trained biostatisticians and quantitative methodology experts as well as subject-matter experts with varied formal training in statistics. In assessing each of these components of a manuscript under review, the reviewer may need to consider additional specialized guidelines and weigh evidence from different sources to best understand and recommend best practices.
